# Comprehensive predictors of drug-resistant epilepsy in MELAS: clinical, EEG, imaging, and biochemical factors

**DOI:** 10.1186/s12883-025-04046-2

**Published:** 2025-02-14

**Authors:** Rui Gao, Lihua Gu, Wenchao Zuo, Pan Wang

**Affiliations:** Department of Neurology, Tianjin Huanhu Hospital, Nankai University, Tianjin, China

**Keywords:** MELAS, Epilepsy EEG, Drug-resistant epilepsy

## Abstract

**Background:**

Seizures are a common but often overlooked manifestation of MELAS. This study aimed to describe the characteristics of seizures in MELAS and to analyze the clinical, electroencephalographic, imaging, and biochemical factors associated with drug-resistant epilepsy.

**Methods:**

A single-center retrospective study was conducted to investigate the clinical characteristics of seizures in MELAS patients. The study collected data on clinical features, muscle biopsy results, genetic testing, seizure symptoms, electroencephalography (EEG), neuroimaging findings, cerebrospinal fluid and blood biochemistry, and the modified Rankin Scale (mRS). We also investigated the correlation between seizure frequency and mRS scores. In addition, we analyzed the risk factors for drug-resistant epilepsy in MELAS.

**Results:**

A total of 37 patients with confirmed MELAS (24 males and 13 females) were included in the study. All patients experienced seizures, with an onset age ranging from 14 to 53 years and a mean of 32 years. These MELAS patients experienced a variety of seizure types, with generalized seizures being the most common. EEG findings revealed background rhythm abnormalities in all patients, and epileptiform discharges were observed in 37.8% of patients during the interictal phase. Status epilepticus (OR 16.499; 95% CI, 1.615–168.557; *P* = 0.018) and elevated resting serum lactate levels (OR 8.594; 95% CI, 1.342–59.733; *P* = 0.024) were identified as independent risk factors for drug-resistant epilepsy. In addition, changes in the seizure frequency at the last follow-up compared to baseline were positively correlated with the mRS score. (*r* = 0.533, *p* < 0.001).

**Conclusion:**

Status epilepticus and elevated resting serum lactate levels were predictive of the development of drug-resistant epilepsy in MELAS. Poor seizure control was significantly associated with increased clinical disability. Early identification of high-risk patients for drug-resistant epilepsy could facilitate the development of more effective treatment plans.

**Supplementary Information:**

The online version contains supplementary material available at 10.1186/s12883-025-04046-2.

## Introduction

MELAS is a maternally inherited mitochondrial disease characterized by genetic, biochemical, and clinical complexities. The most common cause is a single mutation, m.3243 A > G, in the MT-TL1 gene.Approximately 25% of individuals with the m.3243 A > G mutation and over 50% of those diagnosed with MELAS experience seizures [1]. Patients with MELAS may experience different types of seizures, which can lead to status epilepticus. Status epilepticus in MELAS not only leads to neuronal death but also triggers severe complications, including infections, electrolyte imbalances, acid-base disturbances, respiratory and circulatory failure, as well as hepatic and renal dysfunction. These factors collectively contribute to high rates of disability and mortality [2]. Therefore, refractory epilepsy in MELAS is associated with a poor prognosis and often linked to neurodegenerative processes [3].

Research on epilepsy in MELAS patients remains relatively limited.The exact role of seizures in accelerating neurodegenerative processes remains unclear.The factors influencing the course, prognosis, and treatment response of epilepsy in MELAS patients are still not fully understood.To address this gap, we conducted a study to investigate the characteristics of seizures in MELAS, examining the relationships between clinical features, electroencephalography, magnetic resonance imaging, and poor prognosis.Early identification of medication-resistant epilepsy allows clinicians to optimize treatment options while minimizing mitochondrial toxicity. This also facilitates the earlier evaluation of alternative therapies and reduces complications associated with refractory seizures [4].

## Methods

### Study design

This retrospective, single-center study included 37 patients admitted to Tianjin Huanhu Hospital between June 2003 and June 2023. All patients were diagnosed with definite MELAS according to the diagnostic criteria reported by Yatsuga et al. [5]. Patients were followed from the first clinical onset through June 2024.The median follow-up period was 6 years (range, 1 to 21 years). Clinical information for each patient was collected through clinical interviews and assessments.

### The collection of clinical, biochemical, and imaging data

We collected data on patients’ gender, age at first onset, pre-onset and initial symptoms, muscle biopsy findings, genetic testing results, biochemical parameters in cerebrospinal fluid and blood, and neuroimaging findings. Initial onset was defined as the first clinical event accompanied by neuroimaging abnormalities, such as seizures, stroke-like episodes, or severe headaches [5]. Biochemical parameters were collected at the time of initial onset, including lactate levels, creatine kinase (CK), creatine kinase-MB (CK-MB), serum creatinine, hemoglobin, blood lipids, uric acid, blood glucose, and glycated hemoglobin.Imaging data were collected to identify lesion locations on the initial cranial Magnetic Resonance Imaging (MRI) (non-contrast or contrast-enhanced) at disease onset and to monitor brain atrophy and lesion evolution during follow-up MRI. All MRI data were acquired using a Siemens 3.0 Tesla MRI system.

### Collection of seizure data

Age at initial seizure onset, seizure type, antiepileptic drug regimens, and EEG results were collected. At the end of the follow-up period, data on antiepileptic drug therapy, seizure frequency, and drug-resistant epilepsy were documented.Drug-resistant epilepsy was defined as failure of two appropriately chosen and tolerated antiepileptic drugs (either monotherapy or combination therapy) to achieve and maintain sustained seizure freedom [6, 7]. A significant reduction in seizures is defined as a ≥ 50% decrease in seizure frequency from baseline for at least one year. Seizure types were classified according to the ILAE 2017 guidelines [8]. EEG recordings were performed using a Nihon Kohden 7310 16-channel EEG machine under standard clinical conditions.

### Statistical analysis

Based on the definition of drug-resistant epilepsy, the study subjects were classified into drug-resistant and non-resistant groups. The Mann-Whitney U test was used to analyze differences between continuous variables, and Fisher’s exact test to compare categorical variables.Multivariate analysis to identify risk factors for drug-resistant epilepsy was performed using a binary logistic regression model.To assess the potential impact of follow-up duration, a sensitivity analysis was performed, dividing follow-up time into intervals (0–4 years, 4–8 years, 8–12 years, 12–16 years, 16–20 years, and over 20 years) in the multivariable logistic regression analysis. All statistical analyses were performed using SPSS version 29.0, and a p-value of less than 0.05 was considered statistically significant.

## Results

### Clinical characteristics of patients

Clinical information was collected from 37 patients, with a mean follow-up duration of 7.2 ± 4.8 years (range: 1 to 21 years) since their first clinical onset. The mean age of clinical onset for MELAS patients was 32.1 ± 9.8 years (range: 14 to 53 years), and the age at seizure onset was 31.0 ± 10.7 years (range: 12–53). Of the 37 patients, 24 (64.9%) were males.All patients underwent muscle biopsy and genetic testing, with 73% showing ragged red fibers (RRF) and 70.3% showing succinate dehydrogenase strongly reactive blood vessels (SSV). Furthermore, 81.1% of patients had mitochondrial DNA mutations, with all mutations identified as the m.3243 A > G mutation. Hearing loss was the leading symptom before the onset of MELAS, affecting 70.3% of patients, while seizures occurred before MELAS onset in only 21.6% of cases. Seizures were present in all patients at disease onset, with headaches, the most common stroke-like symptom, occurring in 78.4% of cases (Table 1).

**Table 1:** Clinical characteristics of 37 MELAS patients

Clinical Characteristics	Total *n* = 37
Patient(male/female)	37(24/13)
Age of clinical onset(years)	32.1 ± 9.8(14–53)
Age of seizure onset (years)	31.0 ± 10.7(12–53)
Positive family history	28(75.7%)
Muscle biopsy examination	37
RRF	27(73%)
SSV	26(70.3%)
A3243G mutation positive	30(81.1%)
Mutation not found	7(18.9%)
Duration of follow up(years)	7.2 ± 4.8(1–21)
MRS score at last visit	3.5 ± 1.7(1–6)
Clinical manifestations before onset	
Hearing loss	26(70.3)
General fatigue	25(67.6%)
Short stature	24(64.9%)
diabetes mellitus	17(45.9)
Seizure	8(21.6%)
Symptoms at onset	
Seizure	37(100%)
Headache	29(78.4%)
cognitive disorder	24(64.9%)
Status epilepticus	22(59.5%)
Neuropsychiatric disorders	21(56.8%)
Cortical blindness	17(45.9%)
Focal weakness	12(32.4%)
ataxia	10(27%)

### The imaging and biochemical characteristics of 37 MELAS patients

Stroke-like lesions were detected on MRI in all patients, most commonly in the temporal lobe, followed by the occipital lobe. Contrast-enhanced lesions were observed on MRI in 9 patients. Enhanced lesions were found in the parietal lobe (3 patients), occipital lobe (5 patients), temporal lobe (5 patients), dura mater (2 patients), and insular lobe (1 patient). During follow-up, the majority of patients exhibited atrophy or progression of lesions to symmetric areas of the brain. 62.2% of patients developed progressive cerebral atrophy, and 67.6% exhibited bilateral cerebral hemispheric lesions(Table 2). Among the biochemical indicators, most patients showed elevated blood lactate, cerebrospinal fluid lactate, and glycated hemoglobin levels.However, only a small proportion of patients exhibited abnormal levels of CK, CK-MB, creatinine, hemoglobin, triglycerides, cholesterol, fasting blood glucose, or uric acid (Table 3).

**Table 2:** The imaging characteristics of 37 MELAS patients

Image characteristic	Total (*n*%)
Stroke-like lesions in the brain	37(100%)
temporal lobe	31(83.8%)
occipital lobe	25(67.6%)
parietal lobe	24(64.9%)
frontal lobe	8(21.6%)
insular lobe	1(2.7%)
cerebellum	1(2.7%)
Follow-up bilateral lesions	25(67.6%)
Follow-up cerebral atrophy	23(62.2%)
Follow-up cerebellar atrophy	7(18.9%)
The MRI-enhanced lesions	9(24.3%)

**Table 3:** The biochemical indicators of 37 MELAS patients

assay index	Subset	Total(*n*%)
blood lactate(mmol/L)	>2	20(54.1%)
Cerebrospinal fluid lactic acid(mmol/L)	>2.2	35(94.6%)
CK(UL)	>310/200^a^	11(29.7%)
Ckmb(UL)	>24	11(29.7%)
Creatinine(umol/L)	>97/73^b^	3(8.1%)
Hemoglobin(g/L)	≤ 130/115^c^	12(32.4%)
Triglyceride(mmol/L)	>1.7	12(32.4%)
cholesterol(mmol/L)	>5.18	6(16.2%)
Fasting blood glucose(mmol/L)	>6.1	15(40.5%)
uric acid(umol/L)	>420/350^d^	6(16.2%)
glycated hemoglobin(%)	>6	19(51.4%)

a: male310,female200;b: male97,female73;c: male130,female115,All hemoglobin values were below the upper limit of normal; d male420,female350

### Epileptic characteristics of 37 MELAS patients

Generalized seizures were the most common type, occurring in 75.7% of cases, with tonic-clonic seizures accounting for 64.9% of them. Focal seizures were observed in 67.6% of patients, with clonic seizures being the most frequent type, occurring in 43.2% of those cases.One case of focal epilepsy was associated with impaired consciousness, presenting as focal impaired awareness seizures. Sixteen patients experienced both focal and generalized seizures during the course of the disease.All 37 patients exhibited abnormal EEG background rhythms, with generalized background slowing being the most common, observed in 70.3% of cases. Interictal epileptic discharges were observed on EEG in 14 patients, including 10 with focal epileptic waves and 4 with multifocal epileptic waves. All patients received anti seizure medication at the onset of seizures, and high-toxicity antiepileptic drugs such as valproic acid, carbamazepine, phenytoin, and phenobarbital were avoided.The maximum number of antiepileptic drugs used was 1.8 ± 0.78 (range: 1–3), while the number at the final follow-up was 1.4 ± 0.95 (range: 0–3). Overall, 32.4% of the patients developed drug-resistant epilepsy. By the end of follow-up, 10 of the 37 patients had died. Eight deaths were attributed to seizures, including status epilepticus and complications of epilepsy; one death was due to pulmonary infection, and one to heart failure. Notably, 9 of these patients had drug-resistant epilepsy( Table 4).

**Table 4:** Epileptic characteristics of 37 MELAS patients

Epileptic characteristics	*n*(%)
Seizure type	
Focal onset	25(67.6%)
Motor seizures	21(56.8%)
myoclonic seizure	2(5%)
clonic seizure	16(43.2%)
automatism	1(3%)
tonic seizure	2(5%)
Nonmotor seizures	4(10.8%)
Sensory seizure	2(5%)
Cognitive seizures	1(3%)
Emotional seizures	1(3%)
Generalized onset	28(75.7%)
Motor seizures	27(73%)
tonic clonic seizure	24(64.9%)
tonic seizure	2(5%)
clonic seizure	1(3%)
Nonmotor seizures	1(3%)
Typical absence seizure	1(3%)
Focal seizures and generalized seizures	16(43.2%)
Electroencephalography	37(100%)
Background rhythm abnormalities	37(100%)
Focal slowing	11(29.7%)
Generalized slowing	26(70.3%)
Interictal epileptiform discharges	14(37.8%)
Focal sharp/spike wave discharges	10(27%)
Multifocal sharp/spike wave discharges	4(10.8%)
Antiepileptic drugs	37(100%)
Maximal number used	1.8 ± 0.78(1–3)
Numbers using at the last visit	1.4 ± 0.95(0–3)
Drug-resistant epilepsy	12(32.4%)
Response at the last visit*	
Complete seizure freedom for at least 1 year	25(67.6%)
Significant reduction in seizures for at least 1 year	4(10.8%)
No change from baseline	8(21.6%)

### Correlation between drug-resistant epilepsy and clinical, imaging, biochemical, and seizure characteristics

To identify risk factors associated with drug-resistant epilepsy in MELAS patients, we divided the cohort into drug-resistant and non-drug-resistant groups.Univariate analysis revealed that patients presenting with status epilepticus at disease onset were more likely to develop drug-resistant epilepsy than those without status epilepticus. Similarly, patients with elevated blood lactate levels had a higher risk of developing drug-resistant epilepsy than those with normal levels (Supplementary Table 1). Multivariate analysis, adjusting for age and sex, indicated that status epilepticus at onset (OR: 16.499, 95% CI: 1.615–168.557, *P* = 0.018) and elevated blood lactate levels (OR: 8.594, 95% CI: 1.342–59.733, *P* = 0.024) were independent risk factors for drug-resistant epilepsy (Supplementary Table 2). We further evaluated the impact of follow-up duration on the results, and sensitivity analysis confirmed the robustness of the primary findings (Supplementary Table 3).

### Correlation between changes in seizure frequency during the last year of follow-up and the mRS score

At the final follow-up, the response to epilepsy treatment was classified into three categories: seizure-free for at least one year, significant reduction in seizure frequency, and no change in seizure frequency from baseline. Correlation analysis showed a significant positive association between the change in seizure frequency during the final year of follow-up and the mRS score at follow-up (*r* = 0.533, *p* < 0.001). More frequent seizures during follow-up were associated with greater disability and poorer quality of life in patients.

## Discussion

MELAS syndrome is one of the most common forms of mitochondrial encephalomyopathy, distinguished by its unique clinical, genetic, and biochemical features [9].Previous studies have shown that epilepsy in MELAS patients exhibits significant heterogeneity in seizure types, frequency, and treatment response [10]. Seizures in MELAS may present as part of stroke-like episodes or occur independently, and in some cases, they may even trigger stroke-like episodes [11].This study explored the associations between epilepsy outcomes and various factors, including clinical phenotypes, MRI findings, and CSF and blood biochemical parameters in MELAS patients. The aim was to better understand the epileptic characteristics and their potential impact on the prognosis of these patients.

Epilepsy is a common clinical manifestation in MELAS, with prevalence rates ranging from 56.3 to 90% in various studies [8]. Previous research has shown that epilepsy in MELAS involves various types of seizures, including focal seizures with or without impaired consciousness, partial status epilepticus(SE), generalized status epilepticus, and focal-to-bilateral tonic-clonic seizures.The incidence of status epilepticus is highest among the various mitochondrial disease subtypes in MELAS patients [12]. A study of nine MELAS patients found generalized tonic-clonic seizures to be the most common (33%) [13]. Similarly, several studies have demonstrated that generalized seizures are the most prevalent in MELAS, followed by focal seizures [9]. Our findings are consistent with previous studies, showing a higher proportion of patients experiencing generalized seizures than focal seizures.Interestingly, among generalized seizures, tonic-clonic seizures were the most common. However, some studies have suggested that focal seizures are the predominant type of epilepsy observed in MELAS patients.For example, a study of 22 adolescent and pediatric MELAS patients found that only 31.8% experienced generalized seizures, while 95.5% had focal seizures.Demarest et al. studied seven patients with the A3243G mutation and concluded that seizures, particularly those associated with stroke-like episodes, were predominantly focal [14].Stroke-like episodes in MELAS may lower the seizure threshold, predisposing patients to prolonged focal seizures [15].Such prolonged focal seizures may lead to varying degrees of cognitive impairment or evolve into secondary generalized seizures [16].

Fujimoto et al. demonstrated the presence of both generalized and focal epileptiform discharges in the chronic phase of MELAS [17].In this study, all patients showed abnormal EEG background rhythms, primarily characterized by generalized slowing.Interictal epileptiform discharges were observed in 37.8% of the patients. MRI and EEG findings showed that focal slowing and epileptiform abnormalities primarily affected the posterior cortical regions [18].No specific EEG characteristics have been identified that can definitively diagnose MELAS.Previous studies have shown that EEGs in MELAS patients may show focal or multifocal epileptiform abnormalities even during asymptomatic periods, or may appear normal [19].Patients with normal EEG findings might have deeper, less detectable seizure foci or may be in a compensatory phase of cerebral ischemia and hypoxia at the time of the examination [1].

Epilepsy in MELAS syndrome is often resistant to conventional antiepileptic treatments [14].In our study cohort, the incidence of drug-resistant epilepsy (DRE) was 32.4%.Previous studies have shown that the risk of developing drug-resistant epilepsy in newly diagnosed pediatric or adult epilepsy patients is approximately 27% [20].In contrast, other research has shown that the proportion of drug-resistant epilepsy in MELAS patients is significantly higher, at 52.6% [21].

The underlying mechanisms for the high incidence of drug-resistant epilepsy in patients with MELAS remain unclear. The coexistence of mitochondrial damage and epilepsy is well established [22].Furthermore, studies have shown that structural brain abnormalities damage the endothelial cells of the blood-brain barrier capillaries, leading to overexpression of efflux transporters and drug resistance, a mechanism known as the transporter hypothesis [23].Some studies suggest that seizures induce structural changes such as axonal sprouting, synaptic reorganization, gliosis, and neurogenesis in the brain, leading to the formation of abnormal neural networks that hinder the penetration of antiepileptic drugs, ultimately resulting in drug-resistant epilepsy [24].Additionally, some studies have indicated that inherent drug resistance, due to genetic variations in proteins involved in the pharmacokinetics and pharmacodynamics of antiepileptic drugs, could also contribute to the mechanisms behind drug-resistant epilepsy [25].

Multivariate analysis identified status epilepticus as an independent risk factor for the development of drug-resistant epilepsy.Prolonged SE can result in pharmacoresistance, particularly to first-line treatments such as benzodiazepines [26]. A meta-analysis has shown that factors such as SE, EEG abnormalities (including slow waves and epileptiform discharges), and the presence of multiple seizure types are significant risk factors for developing DRE in newly diagnosed epilepsy patients [20].

Imaging changes during seizures in MELAS syndrome may include stroke-like episodes, white matter lesions, cortical atrophy, or may appear normal [27].Previous studies have shown that brain atrophy is a poor prognostic indicator in MELAS epilepsy patients, although the underlying mechanism remains unclear [1].A study of 91 patients with convulsive SE found cortical or hippocampal abnormalities on imaging to be independent predictors of progression to DRE following convulsive SE [28].

Additionally, elevated lactate levels were identified as an independent risk factor for refractory epilepsy. Previous studies have found that elevated lactate levels were also present in the serum of children with drug-resistant epilepsy [29].For each 0.1 mmol/L increase in resting serum lactate levels, the likelihood of drug-resistant epilepsy in MELAS patients increases by 8.0% [30].Elevated lactate levels may act as a biomarker for persistent seizures, potentially leading to the development of drug-resistant epilepsy [31].There is a vicious cycle between mitochondrial dysfunction and seizures, with elevated lactate levels indicating impaired mitochondrial oxidation, leading to the generation of reactive oxygen species and neuronal death, which may be associated with seizures [32].In individuals with the m.3243 A > G mutation, heteroplasmy levels in skeletal muscle and blood were associated with disease severity. Serum lactate levels may also reflect the heteroplasmy mutation burden and be related to poor prognosis [30].

This study found that a higher number of seizures during follow-up was associated with higher mRS scores and poorer prognosis.These findings reveal the vicious cycle of mitochondrial damage and seizures in MELAS patients.Earlier research has also shown a positive correlation between mRS scores and the presence of drug-resistant epilepsy in MELAS patients [21].Of the 10 patients who died in this study, 9 were diagnosed with drug-resistant epilepsy.Delaj et al. demonstrated a significant relationship between increased mortality and the presence of refractory epilepsy [33].

MELAS-associated epilepsy is often refractory to treatment, with a limited number of clearly effective drug options available [34].Antiepileptic medications with high mitochondrial toxicity, including sodium valproate and phenobarbital, are associated with adverse outcomes in patients with MELAS-related epilepsy [1, 35].The antiepileptic drugs used in this study included levetiracetam, benzodiazepines, topiramate, oxcarbazepine, and lamotrigine.Due to the small sample size, no significant association between these drugs and epilepsy prognosis was found.Previous studies have suggested that lamotrigine and levetiracetam may have neuroprotective effects [36].Currently, many new therapeutic approaches for mitochondrial diseases are in various stages of development [37].

Our study had some limitations. First, as a retrospective study, our research may have been subject to recall bias.Although follow-ups were conducted face-to-face, and the statistics on epilepsy were based on patients’ medical records and EEG results, inaccuracies could still have occurred. Second, since lactate levels decrease over time following a seizure [38], we consistently collected blood samples shortly after the seizures.However, different types of seizures may have different effects on lactate levels, potentially affecting our measurements.Finally, due to the small sample size of our cohort, no correlation was found between MRI or EEG findings and drug-resistant epilepsy.We were also unable to perform statistical adjustments for certain potential confounding factors.Future studies should involve larger sample sizes and consider longitudinal designs to better identify indicators associated with drug-resistant epilepsy. Regarding our patient cohort, despite being a specialized hospital for neurological conditions, our institution primarily treated adult patients, which influenced the age demographics of the study population.

To sum up, this study found that seizures were highly prevalent among MELAS patients, including both generalized and focal seizures.At the onset of epilepsy, patients who presented with status epilepticus or elevated blood lactate levels were more likely to develop drug-resistant epilepsy.In the future, expanding the sample size will be essential to further investigate the correlation between drug-resistant epilepsy and clinical, EEG, and imaging findings in MELAS patients. This will help better predict the occurrence of drug-resistant epilepsy and provide targeted treatment.Given the high prevalence of EEG abnormalities in MELAS patients, routine EEG evaluations should be included in the initial diagnostic work-up for all MELAS patients, even in the absence of overt clinical signs of epilepsy. This ensures early detection and management of subclinical or developing epileptic activity.

## Electronic supplementary material

Below is the link to the electronic supplementary material.

Supplementary Material 1

## Data Availability

All data generated or analysed during this study are included in this published article.

## Abbreviations

MELAS: Mitochondrial Encephalomyopathy, Lactic Acidosis, and Stroke-Like Episodes
EEG: Electroencephalography
mRS: Modified Rankin Scale
MRI: Magnetic Resonance Imaging
RRF: Ragged Red Fibers
SSV: Succinate Dehydrogenase Strongly Reactive Blood Vessels
CK: Creatine Kinase
CK-MB: Creatine Kinase-MB
DRE: Drug-Resistant Epilepsy
SE: Status Epilepticus
