# Supplementary material for: Comprehensive predictors of drug-resistant epilepsy in MELAS: clinical, EEG, imaging, and biochemical factors
Source: BMC Neurol. 2025 Feb 14;25:64. doi: 10.1186/s12883-025-04046-2 (PMC11827305; doi:10.1186/s12883-025-04046-2)
Supplement: Supplementary file 1 — Supplementary Material 1 [file 12883_2025_4046_MOESM1_ESM.docx]

**Supplementary table 1** Association of clinical, imaging , biochemical and Epileptic characteristics with drug-resistant epilepsy in MELAS

|  | Drug-resistant epilepsy (n = 12) | Non-drug-resistant  group (n = 25) | p value |
| --- | --- | --- | --- |
| Gender, n (male:female) | 12(8/4) | 25（16/9） | 1.000 |
| Age of seizure onset≤18 | 3（25%） | 3（12%） | 0.367 |
| Age of clinical onset≤18 | 1(8.3%) | 2(8.0%) | 1.000 |
| Positive family history,n (%) | 9(75.0%) | 19(76%) | 1.000 |
| Clinical manifestations before onset,n(%) |  |  |  |
| Hearing loss | 9(75.0%) | 17(68.0%) | 1.000 |
| diabetes mellitus | 7(58.3%) | 10(40.0%) | 0.482 |
| Seizure | 5(41.7%) | 3(12.0%) | 0.083 |
| Short stature | 7(58.3%) | 17(68.0%) | 0.716 |
| General fatigue | 7(58.3%) | 18(72.0%) | 0.468 |
| Symptoms at onset,n(%) |  |  |  |
| Headache | 10（83.3%） | 19（76.0%） | 1.000 |
| Seizure | 12(100%) | 25(100%) | e |
| Status epilepticus | 11(91.7%) | 11(91.7%) | **0.011** |
| Cortical blindness | 4（33.3%） | 13（52.0%） | 0.319 |
| Focal weakness | 2（16.7%） | 10（40.0%） | 0.263 |
| cognitive disorder | 9（75.0%） | 15（60%） | 0.476 |
| ataxia | 2（16.7%） | 8（32.0%） | 0.445 |
| Neuropsychiatric disorders | 5（41.7%） | 16（64.0%） | 0.291 |
| SLL on the frontal lobe | 3（25.0%） | 5（20%） | 1.000 |
| SLL on the parietal lobe | 6（50%） | 18（72.0%） | 0.274 |
| SLL on the insular lobe | 1（8.3%） | 0（0%） | 0.324 |
| SLL on the occipital lobe | 7（58.3%） | 18（72.0%） | 0.468 |
| SLL on the temporal lobe | 10（83.3%） | 21（84.0%） | 1.000 |
| SLL on the cerebellum | 1（8.3%） | 0（0%） | 0.324 |
| The MRI-enhanced lesions | 4（33.3%） | 5（20.0%） | 0.432 |
| Follow-up cerebral atrophy | 9（75.0%） | 14（56.0%） | 0.306 |
| Follow-up cerebellar atrophy | 4（33.3%） | 3（12.0%） | 0.167 |
| Follow-up bilateral lesions | 7（58.3%） | 18（72.0%） | 0.468 |
| blood lactate(mmol/L)＞2 | 10(83.3%) | 10(40.0%) | **0.017** |
| Cerebrospinal fluid lactic acid(mmol/L)＞2.2 | 11(91.7%) | 24(96.0%) | 1.000 |
| CK(UL)＞a | 4(33.3%) | 7(28.0%) | 1.000 |
| Ckmb(UL)＞24 | 4(33.3%) | 7(28.0%) | 1.000 |
| Creatinine(umol/L)＞b | 0（0.0%） | 3（12.0%） | 0.537 |
| Hemoglobin(g/L)＜c | 5（41.7%） | 7（28.0%） | 0.468 |
| Triglyceride(mmol/L)＞1.7 | 6（50.0%） | 6（24.0%） | 0.146 |
| cholesterol(mmol/L)＞5.18 | 2(16.7%) | 4（16.0%） | 1.000 |
| Fasting blood glucose(mmol/L)＞6.1 | 3(25.0%) | 12(48.0%) | 0.286 |
| uric acid(umol/L)＞d | 1（6.7%） | 5（20.0%） | 0.641 |
| glycated hemoglobin(%)＞6 | 7（58.3%） | 12（48.0%） | 0.728 |
| Seizure type |  |  |  |
| Focal onset | 7（58.3%） | 18（72.0%） | 0.468 |
| Generalized onset | 11（91.7%） | 17（68.0%） | 0.220 |
| Focal seizures and generalized seizures | 6（50%） | 10（40%） | 0.726 |
| Background rhythm abnormalities | 12（100%） | 25（100%） | e |
| Focal slowing | 2(16.7%) | 9(36.0%) | 0.279 |
| Generalized slowing | 10(83.3%) | 16(64.0%) | 0.279 |
| Interictal epileptiform discharges | 7(58.3%) | 7(28.0%) | 0.146 |
| Focal sharp/spike wave discharges | 5(41.7%) | 5(20%) | 0.240 |
| Multifocal sharp/spike wave discharges | 2(16.7%) | 2(8.0%) | 0.582 |

a:male310,female200;b:male97,female73;c:male130,female115,All hemoglobin values were below the upper limit of normal;d male420,female350;e:No statistics were calculated

**Supplementary table 2** Multivariate analysis of characteristics with drug-resistant epilepsy presence in MELAS.

|  | OR | Confidence interval | p value |
| --- | --- | --- | --- |
| Status epilepticus | 16.499 | 1.615-168.557 | 0.018 |
| blood lactate | 8.594 | 1.342-59.733 | 0.024 |

The model included age, gender, initial status epilepticus, and blood lactate.

**Supplementary table 3** Sensitivity analysis of factors associated with drug-resistant epilepsy

|  | OR | Confidence interval | p value |
| --- | --- | --- | --- |
| Status epilepticus | 16.499 | 1.615-168.557 | 0.018 |
| blood lactate | 8.594 | 1.342-59.733 | 0.024 |

The model included age, gender, initial status epilepticus, blood lactate and follow-up duration.
